# Ginseng polysaccharides prevent mastitis through *Lactobacillus murinus*‐derived deoxycholic acid and TGR5 signaling

**DOI:** 10.1002/imo2.70090

**Published:** 2026-03-03

**Authors:** Zhijie Zheng, Huiying Liu, Sizhe Chen, Jianhui Gu, Sisi Cai, Xiang Liu, Raphaela I. Lau, Yao Zhang, Sunjae Lee, Xiaoyan Wang, Kewei Fan, Xiaole Yin, Hein Min Tun, Renyou Gan, Haichong Wu, Qi Su

**Affiliations:** ^1^ MOA Key Laboratory of Animal Virology Zhejiang University Center for Veterinary Sciences Hangzhou China; ^2^ Microbiota I‐Center (MagIC) Hong Kong China; ^3^ Department of Medicine and Therapeutics, State Key Laboratory of Digestive Diseases, Li Ka Shing Institute of Health Sciences The Chinese University of Hong Kong Hong Kong China; ^4^ Key Laboratory of Tumor Microenvironment and Immune Therapy of Zhejiang Province, The Second Affiliated Hospital Zhejiang University School of Medicine Hangzhou China; ^5^ State Key Laboratory of Plant Genomics, Institute of Genetics and Developmental Biology (IGDB) Chinese Academy of Sciences Beijing China; ^6^ Department of Gastroenterology Ruijin Hospital Affiliated to Shanghai Jiao Tong University School of Medicine Shanghai China; ^7^ School of Life Sciences Gwangju Institute of Science and Technology Gwangju Republic of Korea; ^8^ College of Animal Science and Technology Chongqing Three Gorges Vocational College Chongqing China; ^9^ Fujian Provincial Key Laboratory for the Prevention and Control of Animal Infectious Diseases and Biotechnology College of Life Sciences, Longyan University Longyan China; ^10^ School of Civil and Environmental Engineering Nanyang Technological University Singapore Singapore; ^11^ Channing Division of Network Medicine, Department of Medicine Brigham and Women's Hospital, Harvard Medical School Boston Massachusetts USA; ^12^ The Jockey Club School of Public Health and Primary Care, Faculty of Medicine The Chinese University of Hong Kong Hong Kong China; ^13^ Li Ka Shing Institute of Health Sciences, Faculty of Medicine The Chinese University of Hong Kong Hong Kong China; ^14^ Department of Food Science and Nutrition The Hong Kong Polytechnic University Hong Kong China; ^15^ Research Institute for Future Food The Hong Kong Polytechnic University Hong Kong China

**Keywords:** deoxycholic acid, ginseng polysaccharides, LPS‐induced mastitis, *Lactobacillus murinus*, microbiota‐derived metabolites

## Abstract

Mastitis, a common inflammatory disease of breast tissue, has been linked to gut microbiota dysbiosis; however, evidence establishing a causal relationship and the potential for microbiome‐based therapies remains limited. This study investigated the gut‐mammary gland axis and how the prebiotic candidate ginseng polysaccharide (GP) prevents mastitis. Using Lipopolysaccharide (LPS)‐induced mouse models, we demonstrate that mastitis involves gut dysbiosis contributing to pathogenesis. GP prevented mastitis in a microbiota‐dependent manner. Mechanistically, GP selectively enriches *Lactobacillus* (*L*.) *murinus*, boosting its metabolite deoxycholic acid (DCA) level in the bloodstream and mammary tissue. DCA suppressed inflammation by binding TGR5 (Takeda G‐protein‐coupled receptor 5), activating the cAMP‐PKA (cyclic adenosine monophosphate‐protein kinase A) pathway, and inhibiting NF‐κB/NLRP3 inflammasome. Additionally, combining GP with *L. murinus* enhanced protection, suggesting potential as a synbiotic. Overall, these findings reveal a novel gut‐mammary gland axis where GP acts by enhancing *L. murinus*‐derived DCA and TGR5 signaling, highlighting the therapeutic potential of targeting this axis for mastitis prevention and management.

## Highlights

- Ginseng polysaccharides effectively alleviate Lipopolysaccharide (LPS)‐induced mastitis in mice.
- Ginseng polysaccharides can enrich and promote intestinal colonization of *Lactobacillus murinus*.
- *Lactobacillus murinus*‐derived deoxycholic acid alleviates LPS‐induced mastitis in mice via the TGR5/cAMP‐PKA signaling pathway.
- The combined use of Ginseng polysaccharide and *Lactobacillus murinus* as a synbiotic exerts a potent alleviative effect on LPS‐induced mastitis.

## Graphical abstract

Prebiotic Ginseng polysaccharides (GP) alleviate mastitis through selective enrichment of gut *L. murinus*, which elevates its anti‐inflammatory metabolite deoxycholic acid (DCA). Circulating DCA engages mammary epithelial TGR5 receptors, triggering the cAMP–PKA pathway to suppress NF‐κB/NLRP3‐mediated inflammation. The synergistic action of GP and *L. murinus* underscores the translational promise of this synbiotic strategy. Targeting the gut–mammary axis—specifically the identified microbial (*L. murinus*) and metabolic circuits—offers a compelling, non‐antibiotic approach for mastitis prevention and therapy.

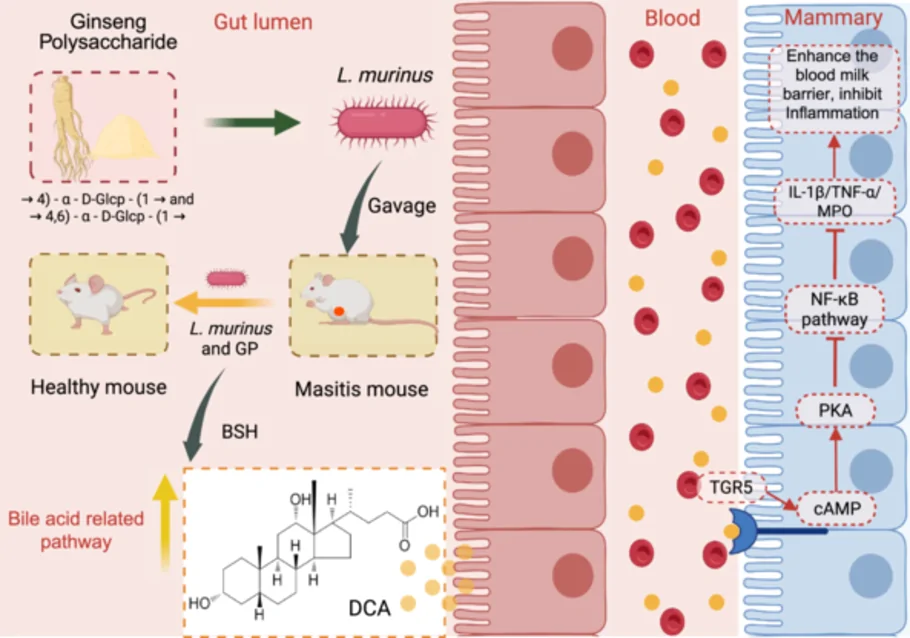

## 1 INTRODUCTION

Preventing mastitis is essential to reduce antibiotic use in lactation, thus lowering the risk of adverse effects on offspring [1, 2, 3]. Growing evidence increasingly indicates a complex relationship between the gut microbiota and mammary health, possibly mediated through a gut–mammary axis involving immune modulation, metabolic signaling, and microbial translocation [4, 5, 6]. Dysbiosis of the gut microbiome is often observed in connection with mastitis, and transferring dysbiotic microbiota from affected cows to mice induced mastitis, suggesting its role in the disease's pathogenesis or progression [7, 8]. Proposed mechanisms include compromised gut barrier integrity that facilitates systemic inflammation, altered immune cell priming in gut‐associated lymphoid tissue impacting mammary immunity, and the direct or indirect effect of microbial metabolites on mammary epithelial cell function and inflammatory pathways [9, 10]. However, while observational associations exist, solid evidence establishing a definitive causal link between specific alterations in gut microbiota and the onset of mastitis is still limited. As a result, the therapeutic potential of modulating the gut microbiome to prevent mastitis, although conceptually promising, remains largely unexplored and needs thorough mechanistic validation.

Prebiotics, such as polysaccharides derived from medicinal herbs, offer a promising approach to selectively modulate the gut microbiome towards a beneficial composition [11, 12, 13]. Ginseng polysaccharide (GP), a key bioactive component of *Panax ginseng*, has demonstrated strong prebiotic effects, including promoting the growth of beneficial bacteria like *Lactobacillus* and *Bifidobacterium spp*., strengthening gut barrier function, and exerting systemic anti‐inflammatory activities [14, 15, 16, 17]. Notably, *Panax ginseng,* as an herbal medicine, has long been traditionally used to treat mastitis [18]. Considering its established role in modulating the gut microbiome and immune regulation, GP is a compelling candidate for microbiome‐targeted interventions against mastitis. Therefore, we hypothesize that GP prevents mastitis by altering the gut microbiota to boost beneficial taxa and their immunomodulatory metabolites, thus harnessing the gut‐mammary axis.

In this study, we examined the gut‐mammary axis in mastitis and how GP offers protection. Our research demonstrates that gut dysbiosis directly causes mastitis. GP fights mastitis by increasing *Lactobacillus murinus* levels, which raises deoxycholic acid (DCA) to activate mammary TGR5/cAMP‐PKA signaling and suppress NF‐κB/NLRP3 inflammation, with enhanced effects seen when combining GP and *L. murinus*. Overall, this study provides causal evidence for the gut‐mammary axis, outlines the GP‐*L. murinus*‐DCA‐TGR5 pathway, and shows strong potential of synbiotic therapy for the therapy of mastitis.

## 2 RESULTS

### Fecal microbiota from mastitis donors promotes mastitis in recipient mice

To explore the causation link between mastitis and gut microbiota, we first established an LPS‐induced mastitis mouse model, confirmed by pathological analysis (Figure 1A, Figure S1A), and then performed 16S rRNA sequencing on colon microbiota. Principal coordinates analysis (PCoA) results showed a clear separation between the control (NC) and mastitis (LPS) groups (Figure 1B), with a statistical significance of *p* = 0.013 and *R*² = 0.3189, alongside significant downregulation of gut homeostasis indicators, including the *Firmicutes/Bacteroidota* ratio and α‐diversity (Figure 1C–E). Microbial composition analysis revealed significant depletion of *Firmicutes* (specifically probiotic *Lactobacillus*) and enrichment of pathogenic *Escherichia‐Shigella* in the LPS group (Figure S1B [genus level]). Overall, these findings suggested that LPS‐induced mastitis is associated with significant gut dysbiosis.

**Figure 1:**
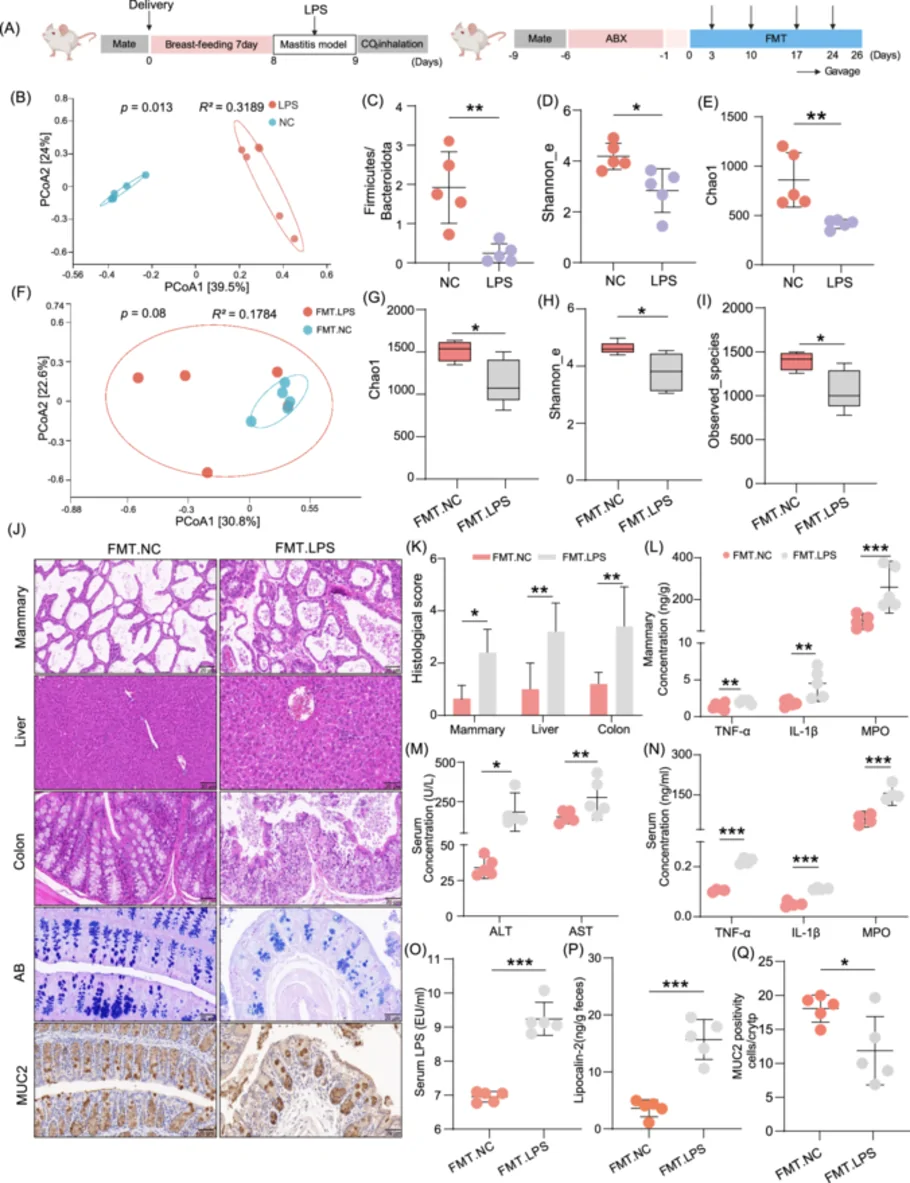
Mastitis‐associated fecal microbiota can induce mastitis in recipient mice. (A) Experimental protocol. After 7 days of breastfeeding in mice. On the 8th day, a mastitis model was established. On the 9th day, the mice were euthanized. For fecal microbiota transplantation (FMT), mice were mated for 3 days. After confirming pregnancy, these mice were treated with an antibiotic cocktail for 5 days to deplete the commensal microbiota and performed FMT for 26 days. (B) Principal coordinates analysis (PCoA) analysis was conducted to assess the compositional shifts in gut microbiota between NC and Lipopolysaccharide (LPS) group mice (*p* = 0.013, *R*² = 0.3189, *F* = 3.746). (C–E) *Firmicutes*/*Bacteroidota* ratio, Shannon_e and Chao1 index of the gut microbiota were compared between NC and LPS groups (*n* = 5). (F) PCoA analysis was conducted to evaluate changes in gut microbiota composition between NC and FMT.LPS group mice (*p* = 0.08, *R*² = 0.1784, *F* = 1.3027). (G–I) Observed species and α‐diversity indices (including Shannon_e and Chao1) were measured in NC and FMT.LPS groups (*n* = 5). (J) Representative H&E‐stained sections of mammary, liver, and colon tissues, as well as the colonic mucous layer stained with Alcian blue (AB), were analyzed (scale bar, 20 μm; *n* = 5). Immunohistochemistry using a mucus‐2 (MUC2) antibody revealed brownish‐yellow positive cells (*n* = 5). (K) Histological scoring of mammary, liver, and colon tissues was based on H&E‐stained sections (*n* = 5). (L) Mammary IL‐1β, TNF‐α, and MPO concentrations were detected by ELISA kit (*n* = 5). (M, N) Serum ALT (Alanine Aminotransferase) and AST (Aspartate Aminotransferase) activities and serum IL‐1β, TNF‐α, and MPO concentrations were detected in each group (*n* = 5). (O, P) Fecal Lipocalin‐2 and Serum LPS concentrations were measured by ELISA kit (*n* = 5). (Q) The number of MUC2‐positive cells per crypt was quantified (*n* = 5). All data are presented as the mean ± standard error of the mean (SEM). For comparisons between two groups, statistical significance was assessed using an unpaired two‐tailed Student's *t*‐test. For comparisons among three or more independent groups in a single‐factor design, a one‐way analysis of ANOVA was performed. For non‐parametric comparison of histological scores among multiple groups, the Kruskal–Wallis test was applied. Statistical significance was indicated as follows: *p < 0.05, **p < 0.01, ***p < 0.001. ANOVA, one‐way analysis of variance.

We then transplanted fecal microbiota from LPS‐induced mastitis mice (FMT.LPS) or healthy mice (FMT.NC) into pseudo‐germ‐free (ABX‐treated) recipients to test whether gut dysbiosis directly causes mastitis (Figure 1A). Microbiota analysis confirmed the efficacy of antibiotic treatment, revealing significantly reduced α‐ and β‐diversity in ABX‐treated mice (A‐NC) compared to NC mice before FMT (Figure S1C–F). Post‐FMT, PCoA analysis demonstrated distinct microbial community structures between the FMT.LPS and FMT.NC groups (Figure 1F), with a statistical significance of *p* = 0.08 and *R*² = 0.1784. Consistently, the FMT.LPS group exhibited significantly lower α‐diversity and observed species compared to the FMT.NC group (Figure 1G–I). Moreover, it showed downregulation of *Lactobacillus* (Figure S1G [genus level]), confirming the pronounced gut dysbiosis induced by FMT.LPS.

Furthermore, we evaluated mammary gland histopathology and inflammation. FMT.LPS recipients displayed significantly exacerbated mammary gland injury and inflammatory responses compared to FMT.NC recipients (Figure 1J–K). Western blotting revealed markedly reduced levels of blood‐milk barrier tight junction (TJ) proteins (ZO‐1, Occludin, Claudin‐3) in the FMT.LPS group (Figure S1H,I) [19]. Given the critical role of the NF‐κB/NLRP3 pathway in mastitis pathogenesis [20]. We next evaluated key proteins involved in this pathway. FMT.LPS significantly upregulated expression of NLRP3, p‐p65, and p‐IκBα (Figure S1H,I), suggesting dysbiosis exacerbates mastitis via this pathway.

To further elucidate the underlying mechanisms by which gut microbiota dysbiosis induces mastitis, we measured the impact of FMT.LPS on the immune system and intestinal barrier in the recipient mice. FMT.LPS recipients showed severe histopathological damage in both colon and liver tissues (Figure 1J,K). Pro‐inflammatory cytokine levels in the mammary gland were also found to be elevated in FMT.LPS recipients, while serum analysis further revealed significantly increased concentrations of pro‐inflammatory cytokines (IL‐1β, TNF‐α), myeloperoxidase (MPO), lipopolysaccharide (LPS), and liver enzymes (ALT, AST, Figure 1L–O) [21]. Lipocalin‐2, a biomarker of intestinal mucositis, was significantly elevated in the FMT.LPS group compared to the FMT.NC group (Figure 1P). This evidence confirmed the severe damage in both the colon and liver caused by gut dysbiosis.

The intestinal barrier serves as the first line of defense against commensal and pathogenic microorganisms, maintaining host immune homeostasis [22]. The reduction in the number of goblet cells and the decrease in the mucin they secrete in the colon tissue are considered one of the signs of colonic injury [23]. We used Periodic Acid‐Schiff and Alcian Blue Stain staining and Mucin 2 (MUC2) to assess the status of goblet cells in the colonic crypts [24, 25]. Results revealed a decrease in the number of goblet cells in the colon of FMT.LPS group, and a corresponding decrease in the production of mucin by goblet cells (Figure 1J,Q). Furthermore, the expression levels of key tight junction proteins such as ZO‐1, Occludin, and Claudin‐3 in the colon of FMT.LPS mice were markedly reduced compared with FMT.NC mice (Figure S1J–K). These findings demonstrate that FMT from mastitis mice into recipient mice disrupts the intestinal barrier, systemic inflammation, and ultimately contributes to the occurrence of mastitis.

### Structural characterization of GP

In the present study, an array of methodologies was employed to characterize the structural and compositional features of GP. FTIR analysis revealed characteristic polysaccharide absorption bands, including O–H and C–H stretching vibrations and signals indicative of a pyranose ring structure (Figure S2A). Furthermore, SEM imaging showed that GP possesses a rough, porous, and irregular surface morphology, consistent with typical polysaccharide architecture (Figure S2B). Monosaccharide composition analysis demonstrated that GP is primarily a glucan, consisting mainly of glucose with minor amounts of mannose and galactose (molar ratio 0.311:0.701:98.998) (Figure S2C,D).

### Analysis of glycosidic bonds in GP

The results of the main glycosidic bonds found in GP are shown in Table S2. The results shown in the table indicate that the main polysaccharide sequences in GP are composed of t‐Glcp, 4‐Glcp, 6‐Glcp, and 4,6‐Glcp, with a relative molar ratio of 16.39:74.12:0.89:8.60. In addition, among all glycosidic bonds, 4‐Glcp is the largest sugar residue (74.12%), indicating that it may be the main form of GP.

### NMR analysis

Comprehensive structural analysis, integrating monosaccharide composition, methylation data, and multi‐dimensional NMR spectroscopy (¹H‐NMR, ¹³C‐NMR, COSY, HSQC, HSQC‐TOCSY, HMBC, NOESY), revealed that GP is a glucan primarily composed of α‐d‐glucopyranose (Glcp) residues (Figure S2E–K).

Methylation analysis indicated the main linkage types were →4) ‐α‐d‐Glcp‐ (1→ (74.12%), →4,6) ‐α‐d‐Glcp‐ (1→ (8.60%), and terminal α‐d‐Glcp, with a minor proportion of 1,6‐linked Glcp. These assignments were confirmed by characteristic anomeric signals in the HSQC spectrum (e.g., δH/δC 5.27/99.44 ppm for residue A [→4) ‐α‐d‐Glcp‐ (1→] and 5.23/99.55 ppm for residue B [→4,6) ‐α‐d‐Glcp‐ (1→]). Downfield shifts of the corresponding carbon atoms (C‐1, C‐4, and C‐6) supported substitution at these positions.

Inter‐residue connectivity was established using HMBC and NOESY spectra. Key correlations (e.g., A H‐1/A C‐4, A H‐1/B C‐4, B H‐1/A C‐4) and corresponding NOE contacts demonstrated that the backbone consists predominantly of →4)‐α‐d‐Glcp‐(1→4)‐α‐d‐Glcp‐(1→ repeats, with →4,6)‐α‐d‐Glcp‐(1→ serving as branch points at approximately every tenth residue.

In summary, GP is an α‐glucan with a backbone of (1→4) ‐linked α‐d‐Glcp units, partially branched at the O‐6 position, as depicted in Figure S2L.

### Oral GP prevents LPS‐induced mastitis via modulation of the gut microbiota

Since preventing mastitis holds greater clinical significance than treatment, we investigated the potential preventive effects of GP in LPS‐induced mastitis. We first characterized the structure of the ginseng polysaccharides used in our study. We first conducted a detailed structural analysis of the ginseng polysaccharides used in our research. The findings revealed that the ginseng polysaccharide (GP) we employed is predominantly a glucan, characterized by a glucose‐rich backbone (Figure S2). This finding demonstrates that the structure of the ginseng polysaccharide we employed is representative of its class [26, 27]. We then established experimental mastitis in mice (Figure 2A). Compared to the PBS group, LPS challenge induced severe mastitis characterized by mammary tissue injury, inflammatory cell infiltration, and heightened inflammatory responses. GP treatment significantly inhibited LPS‐induced mastitis in a dose‐dependent manner (Figure 2B) and suppressed pro‐inflammatory cytokine production (MPO, TNF‐α, IL‐1β; Figure S3A). Notably, its therapeutic efficacy is on par with that of the widely used clinical drug dexamethasone (DEX) [28]. The blood‐milk barrier, primarily composed of tight junction (TJ) proteins, is critical for defense against pathogens [29]. Following LPS challenge, GP preconditioning effectively restored mammary epithelial barrier integrity, evidenced by the upregulated expression of TJ proteins (ZO‐1, Occludin, Claudin‐3) compared to the LPS‐treated group (Figure S3B,C). The LPS‐induced inflammatory cytokine surge is driven by activation of pro‐inflammatory pathways like NF‐κB/NLRP3, central to mastitis pathogenesis [30, 31]. Western blot analysis revealed that GP pretreatment potently inhibited LPS‐induced activation of both NLRP3 and NF‐κB pathways (Figure S3D,E).

**Figure 2:**
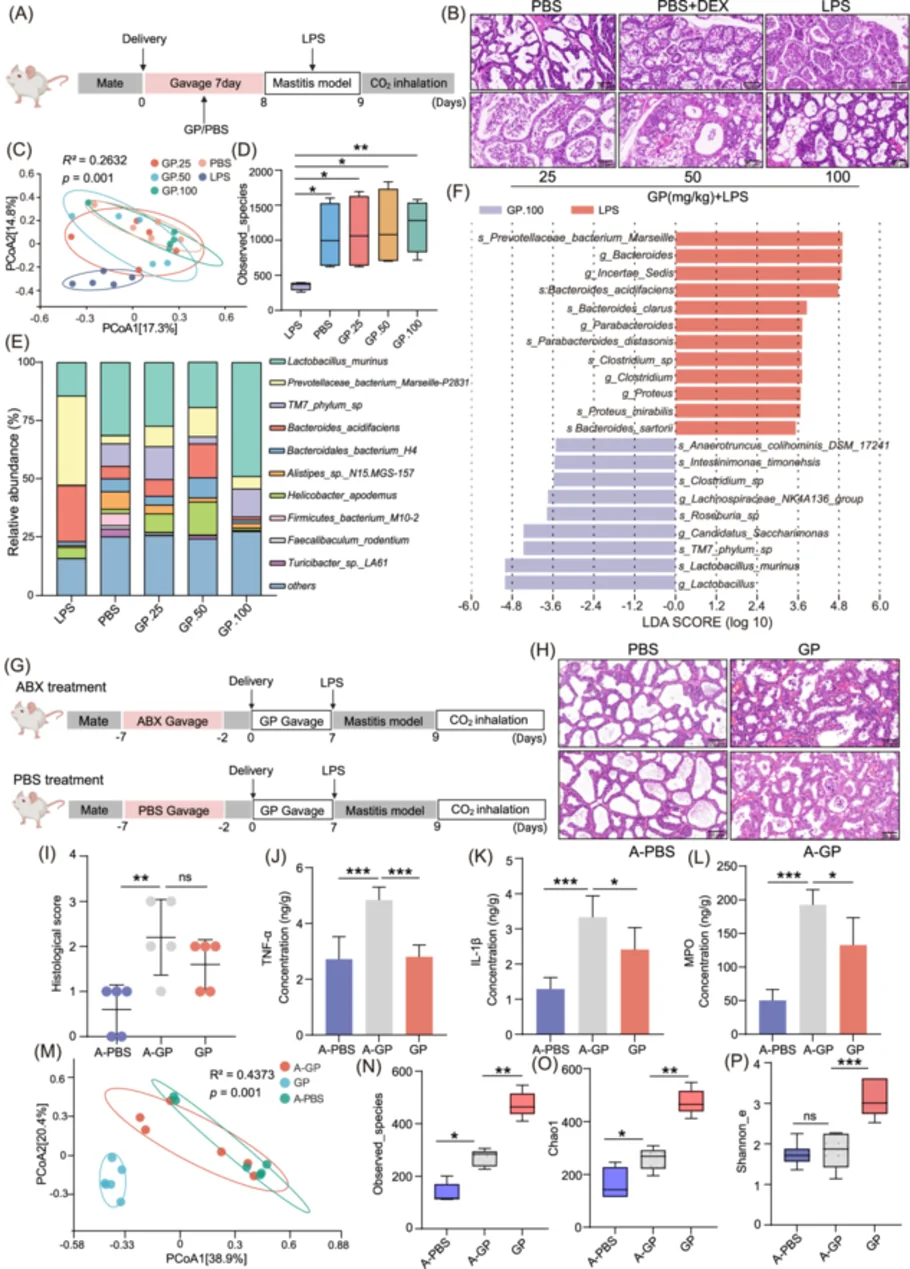
Oral GP prevents LPS‐induced mastitis via modulation of the gut microbiota. (A) Experimental protocol. Mice were administered gavage with different concentrations of GP for 7 days after parturition. On the 8th day, a mastitis model was established. On the 9th day, the mice were euthanized. (B) Representative H&E staining images of mammary gland. Scale bars: 20 μm. (C) Principal coordinates analysis (PCoA) analysis was conducted to measure the changes in the composition of the gut microbiota in each group (*p* = 0.001, *R*² = 0.2632, *F* = 1.7859). (D) The observed species were measured in each group (*n* = 5). (E) The bacterial composition at the species level was examined for the indicated groups. (F) LEfSe analysis was conducted to identify the different bacterial taxa enriched in different groups (log_10_LDA score > 3 and FDR < 0.05). (G) Experimental protocol. (H) Representative H&E staining images of mammary gland. Scale bars: 20 μm. (I) Mammary gland histological scores were determined based on H&E‐stained sections (*n* = 5). (J–L) MPO, IL‐1β, and TNF‐α in the mammary gland were measured by ELISA kit (*n* = 5). (M) PCoA analysis was conducted to measure the changes in the composition of the gut microbiota in GP, A‐GP and A‐PBS (*n* = 5). (N–P) α‐diversity indices (including observed species, Chao1 and Shannon_e) were measured in GP, A‐GP and A‐PBS groups (*n* = 5). All data are presented as the mean ± standard error of the mean (SEM). For comparisons between two groups, statistical significance was assessed using an unpaired two‐tailed Student's *t*‐test. For comparisons among three or more independent groups in a single‐factor design, a one‐way analysis of variance (ANOVA) was performed. For non‐parametric comparison of histological scores among multiple groups, the Kruskal–Wallis test was applied. Statistical significance for all tests was indicated as follows: *p < 0.05, **p < 0.01, ***p < 0.001. LPS, Lipopolysaccharide.

To assess GP's impact on gut microbiota, we performed 16S rRNA gene sequencing on colon samples from each mouse group (*n* = 5). PCoA showed distinct clustering separating the GP‐treated and PBS‐treated groups (Figure 2C), with a statistical significance of *p* = 0.001 and *R*² = 0.2632. GP intervention effectively inhibited the LPS‐induced decline in α‐diversity metrics, including observed species (Figure 2D) and Shannon and Chao1 indices (Figure S3G,H). At the phylum level, GP treatment significantly increased the relative abundance of *Firmicutes* (Figure S3F,I), leading to a rebound in the *Firmicutes/Bacteroidota* ratio (Figure S3J). Species‐level analysis identified *Lactobacillus murinus* as the most significantly increased species following GP treatment (Figure 2E [species level]). LEfSe (Linear discriminant analysis Effect Size) analysis corroborated genus‐ and species‐level enrichments in GP‐treated mice, highlighting *Lactobacillus* (genus) and *L. murinus* (species, most prominent) among the increased taxa (Figure 2F).

These findings indicate that GP counteracts LPS‐induced gut microbiota dysbiosis associated with mastitis. We therefore hypothesized that GP's mitigation of mammary gland damage is mediated via gut microbiota modulation. To test this, we employed an antibiotic cocktail to deplete the gut microbiota, creating a pseudo‐germ‐free model, followed by GP treatment using the same protocol (Figure 2G). Strikingly, in antibiotic‐treated mice, the previously observed therapeutic effects of GP—reduction in tissue inflammation and inflammatory cytokines—were abolished (Figure 2H–L). 16S rRNA sequencing confirmed that antibiotic depletion prevented GP from restoring gut microbial diversity or compositional structure (Figure 2M–P). Collectively, these results demonstrate that gut microbiota is essential for mediating GP's protective effects against mastitis.

### GP modulates gut microbiota to increase secondary bile acid DCA production

To explore potential metabolic alterations underlying GP's effects, we performed untargeted metabolomics on fecal samples from the LPS and GP.100 groups, detecting 1396 metabolites. Principal component analysis (PCA) revealed distinct clustering between the LPS and GP.100 groups (Figure 3A). Differential metabolite analysis (FC ≥ 2 or FC ≤ 0.5, *p* < 0.05) identified 75 significantly altered metabolites (29 upregulated, 46 downregulated) in the GP.100 group versus LPS (Figure 3B). Orthogonal partial least squares discriminant analysis with Variable Importance in Projection > 1 refined this to 25 key metabolites: 14 downregulated (e.g., 3,4‐dihydroxyphenylacetic acid) and 11 upregulated (e.g., deoxycholic acid [DCA]) (Figure 3C).

**Figure 3:**
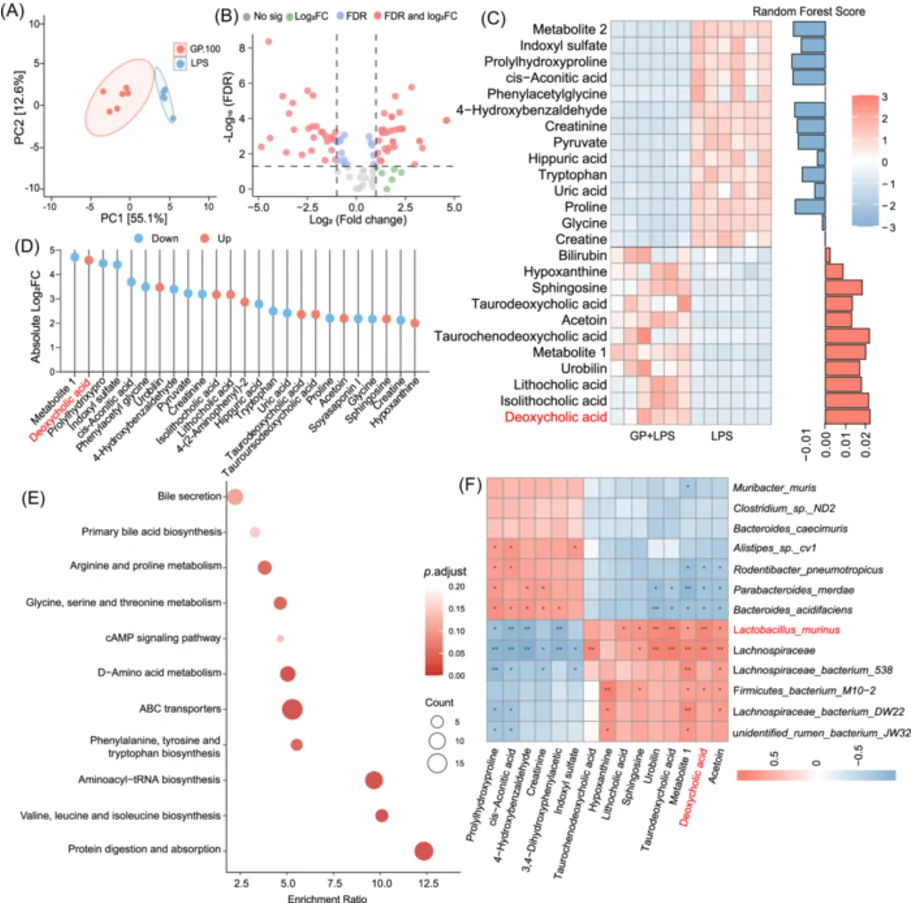
Metabolic profiles of mice gut samples from Lipopolysaccharide (LPS) group and GP.100 group. (A) PCA score plots for feces samples (*n* = 6). (B) Volcano plots show the results of a pairwise comparison of metabolites between the GP.100 and LPS group. The vertical and horizontal dashed lines indicate the threshold for a twofold abundance difference and the threshold for FDR = 0.05, respectively. Significant metabolites appear in red (FDR < 0.05, and FC (Fold Change) ≥ 2 or FC ≤ 0.5). (C) Differential metabolite heatmaps and random forest scores between GP.100 and LPS groups, the intensity of the color represents the degree of change, with blue indicating downregulation and red indicating upregulation. (D) Absolute Log_2_FC value plot of differential metabolites was shown in a bar chart. (E) KEGG pathway. (F) Spearman's rank correlation analysis was applied to assess the pairwise relationships between gut microbiota and differential metabolites in the GP.100 and LPS groups, where statistical significance (marked as * and **) was determined by FDR < 0.05 and FDR < 0.01. Red indicates a positive correlation, while blue indicates a negative correlation. Metabolite 1:4‐(2‐Aminophenyl)‐2,4‐dioxobutanoate, Metabolite 2: 3,4 Dihydroxyphenylacetic acid. One‐way ANOVA with Tukey's test was used for multiple comparisons. ANOVA, one‐way analysis of variance; PCA, principal component analysis.

Among the upregulated metabolites, the secondary bile acid DCA exhibited the highest fold change and ranked the highest in the random forest model for discriminating between groups (Figure 3C,D). KEGG pathway enrichment confirmed bile secretion and Primary bile acid biosynthesis, and cAMP signaling pathway enriched following GP treatment (Figure 3E).

Since *L. murinus* (identified previously as GP‐enriched) contributes to primary‐to‐secondary bile acid conversion [32], we assessed its relationship with DCA. Spearman analysis confirmed a strong positive correlation between *L. murinus* abundance and fecal DCA levels (Figure 3F). Collectively, these results suggest that GP modulates gut health and immune responses, at least partially, through restructuring the gut microbiota (*L. murinus*) and elevating the microbially derived metabolite DCA.

### *L. murinus*‐derived DCA alleviates LPS‐induced mastitis

Based on our identification of *L. murinus* as a key mediator of GP's beneficial effects on mastitis, we directly assessed its therapeutic potential in LPS‐induced mastitis. Oral administration of *L. murinus* significantly inhibited LPS‐induced histopathological damage in mammary tissue and reduced pro‐inflammatory cytokine levels (Figure 4A–C). This intervention also increased intestinal *L. murinus* abundance, and crucially, *L. murinus* treatment significantly elevated DCA levels not only in feces but also systemically in serum and locally within mammary gland tissue (Figure 4D,E), suggesting its role in modulating host DCA distribution.

**Figure 4:**
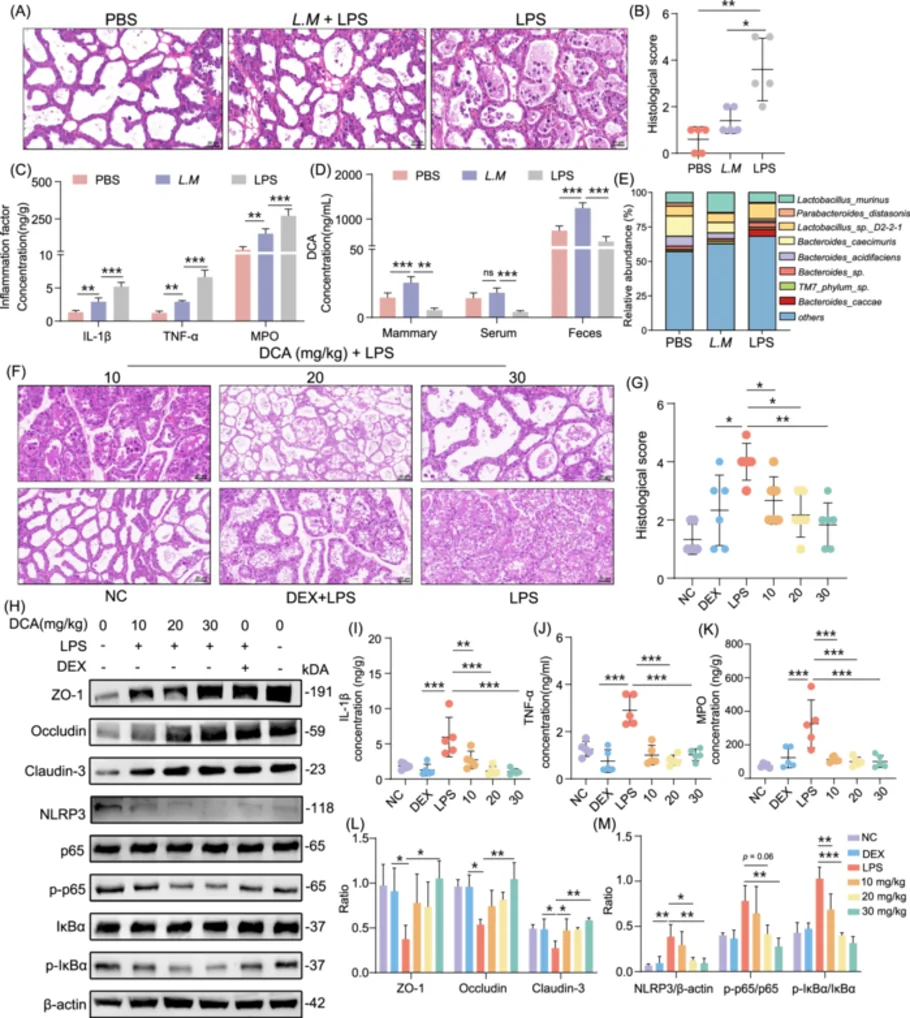
DCA derived from *L. murinus* mitigates LPS‐induced mastitis. (A) Representative H&E staining images of mammary gland. Scale bars: 20 μm. (B) Mammary gland histological scores were determined based on H&E‐stained sections (*n* = 5). (C) MPO, IL‐1β, and TNF‐α in the mammary gland were measured by ELISA kit (*n* = 5). (D) DCA concentration in the mammary gland, feces, and serum was measured by ELISA kit. (E) Bacterial composition at the species level for the indicated groups was examined (*n* = 5). (F) Representative H&E staining images of mammary gland. Scale bars: 20 μm. (G) Mammary gland histological scores were determined based on H&E‐stained sections (*n* = 5). (H) Representative western blot images of NF‐κB/NLRP3 pathway and tight junction (TJ) proteins in mammary gland. (I–K) MPO, IL‐1β, and TNF‐α in the mammary gland were measured by ELISA kit (*n* = 5). (L, M) Relative expression levels of proteins were analyzed using Image J (*n* = 3). All data are presented as the mean ± SEM. For comparisons among three or more independent groups in a single‐factor design, a one‐way ANOVA was performed. For non‐parametric comparison of histological scores among multiple groups, the Kruskal–Wallis test was applied. Statistical significance was indicated as follows: *p < 0.05, **p < 0.01, ***p < 0.001. ANOVA, one‐way analysis of variance; DCA, deoxycholic acid; LPS, Lipopolysaccharide.

These findings strongly indicate that *L. murinus* alleviates mastitis, likely mediated by DCA. To directly test DCA's protective role, mice were pretreated with DCA (10, 20, 30 mg/kg) prior to LPS challenge. DCA dose‐dependently improved mammary gland injury and reduced pro‐inflammatory factors (Figure 4F–K). Furthermore, DCA pretreatment restored the expression of blood‐milk barrier tight junction proteins (ZO‐1, Occludin, Claudin‐3; Figure 4H,L) and suppressed LPS‐induced activation of the NF‐κB/NLRP3 inflammatory pathway (Figure 4H,M).

### DCA relieved LPS‐induced mastitis via TGR5‐cAMP‐PKA‐NF‐κB/NLRP3 signaling pathway

Next, we utilized mouse mammary epithelial cells (MMECs) to elucidate the cellular mechanism by which DCA protects against mastitis (Figure 5A). Screening of bile acid receptors revealed that DCA (30 µM) selectively increased TGR5 expression in MMECs (Figure 5B), with minimal effect on FXR or other receptors [33, 34]. Treatment with DCA reduced LPS‐induced mRNA expression of pro‐inflammatory cytokines (TNF‐α, IL‐1β, IL‐6) in MMECs (Figure 5C). To further define the role of TGR5, we suppressed its expression using *Tgr5* siRNA and inhibited its downstream signaling using cAMP inhibitor MDL12330A and PKA inhibitor H89 [35]. Both silencing *Tgr5* and inhibiting cAMP/PKA signaling reversed the anti‐inflammatory effect of DCA in LPS‐treated MMECs (Figure 5D,E). Taken together, these results demonstrate that TGR5 activation is essential for the anti‐inflammatory effect of DCA.

**Figure 5:**
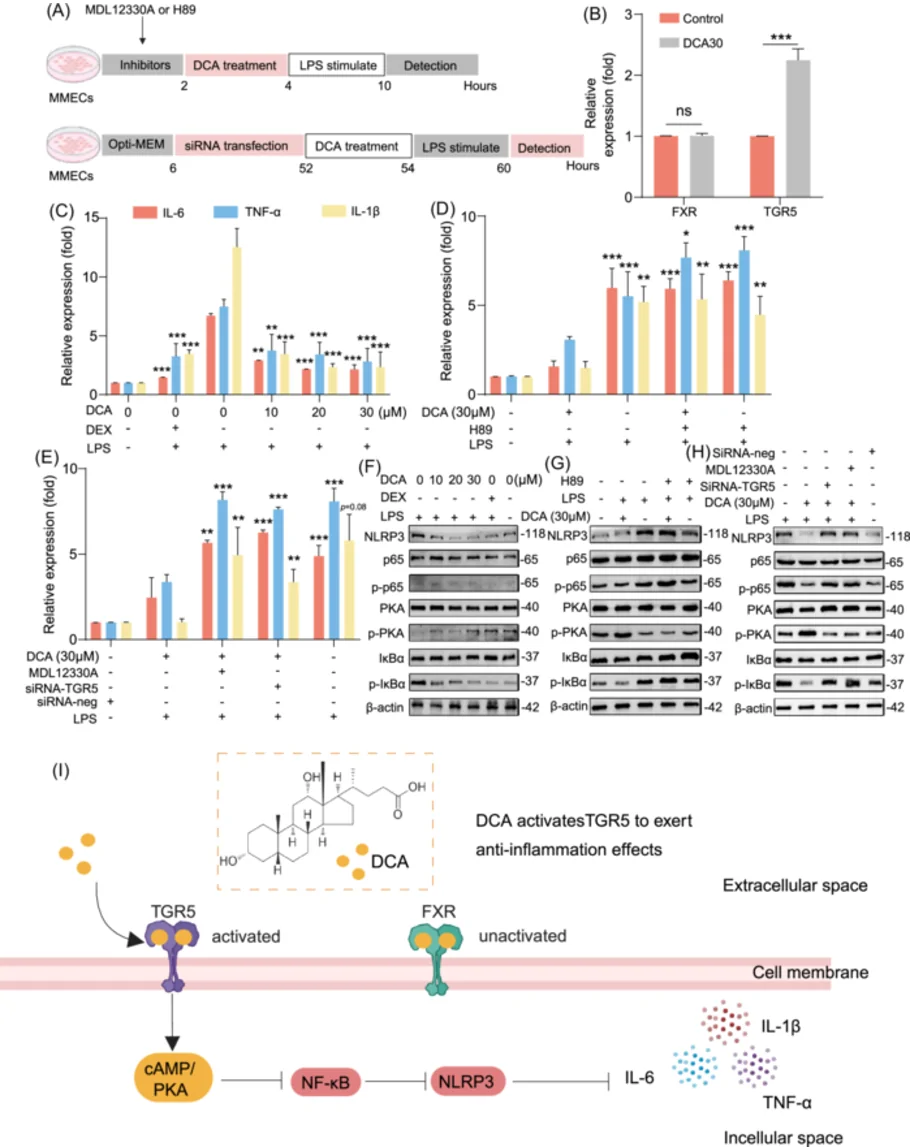
DCA relieved Lipopolysaccharide (LPS)‐induced inflammation in MMECs by activating TGR5. (A) Experimental protocol. For the DCA treatment, MDL12330A (10 μM) or H89 (30 μM) was added 2 h prior to DCA. After 2 h of DCA treatment, LPS was added, and cells were collected after an additional 6 h. For the siRNA experiments, cells were treated with Opti‐MEM for 6 h, followed by siRNA transfection for 48 h. Subsequently, cells were treated with DCA for 2 h, then LPS was added, and cells were collected after an additional 6 h. (B) Cells were treated with DCA (30 μM), and the relative mRNA levels of TGR5 and FXR were determined by RT‐qPCR. (C) Cells were pretreated with DCA (10, 20, and 30 μM) and mRNA levels of pro‐inflammatory related factors IL‐1β, IL‐6, and TNF‐α were detected by RT‐qPCR. The statistical differences of all groups were compared with the LPS group. (D) Cells were pretreated with H89, and the relative mRNA levels of pro‐inflammatory IL‐1β, IL‐6, and TNF‐α were detected by RT‐qPCR. The statistical differences of all groups were compared with the DCA treatment group. (E) Cells were pretreated with *Tgr5* siRNA, MDL12330A, and DCA, and the relative mRNA levels of pro‐inflammatory IL‐1β, IL‐6, and TNF‐α were detected by RT‐qPCR. The statistical differences of all groups were compared with the DCA treatment group. (F–H) Protein levels of cAMP‐PKA‐NF‐κB/NLRP3 pathway were detected by Western blotting in indicating groups. (I) Mechanism diagram of DCA alleviating LPS‐induced MMECs inflammation. All data are presented as the mean ± SEM. For comparisons between two groups, statistical significance was assessed using an unpaired two‐tailed Student's *t*‐test. For comparisons among three or more independent groups in a single‐factor design, a one‐way ANOVA was performed. Statistical significance was indicated as follows: *p < 0.05, **p < 0.01, ***p < 0.001. ANOVA, one‐way analysis of variance.

Given the central role of NF‐κB/NLRP3 signaling in mastitis, we confirmed that DCA dose‐dependently inhibited LPS‐induced activation of this pathway in MMECs (Figure 5F, Figure S4A–D). Since TGR5 signals primarily via the cAMP‐PKA cascade to exert anti‐inflammatory effects [36], we investigated whether this mediates DCA's inhibition of NF‐κB/NLRP3. Using MDL12330A and H89, we found that blocking either cAMP or PKA activity reversed DCA's suppression of NF‐κB/NLRP3 pathway activation (p‐p65, p‐IκBα, NLRP3) and downstream PKA phosphorylation (p‐PKA) in LPS‐challenged MMECs (Figure 5G–H, Figure S4E–L).

Collectively, these results establish that DCA alleviates LPS‐induced inflammation in mammary epithelium by activating TGR5, subsequently triggering the cAMP‐PKA signaling cascade, which ultimately suppresses the pro‐inflammatory NF‐κB/NLRP3 pathway (Figure 5I).

### The combination of GP and *L. murinus* functions as a synbiotic to alleviate LPS‐induced mastitis

Building on the established therapeutic efficacy of both GP and *L. murinus* against mastitis, we hypothesized that their combination would function synergistically as a synbiotic. This hypothesis was tested in both LPS‐induced SPF and pseudo‐germ‐free (PGF) mouse models. The combined treatment significantly alleviated mastitis symptoms, showing superior reduction of mammary inflammation compared to individual treatments (Figure 6A, Figure S5A,B). We further demonstrated in vitro that GP (250–1000 µg/mL) robustly promotes the proliferation of *L. murinus* using MRS medium [37], supporting its prebiotic function (Figure 6B).

**Figure 6:**
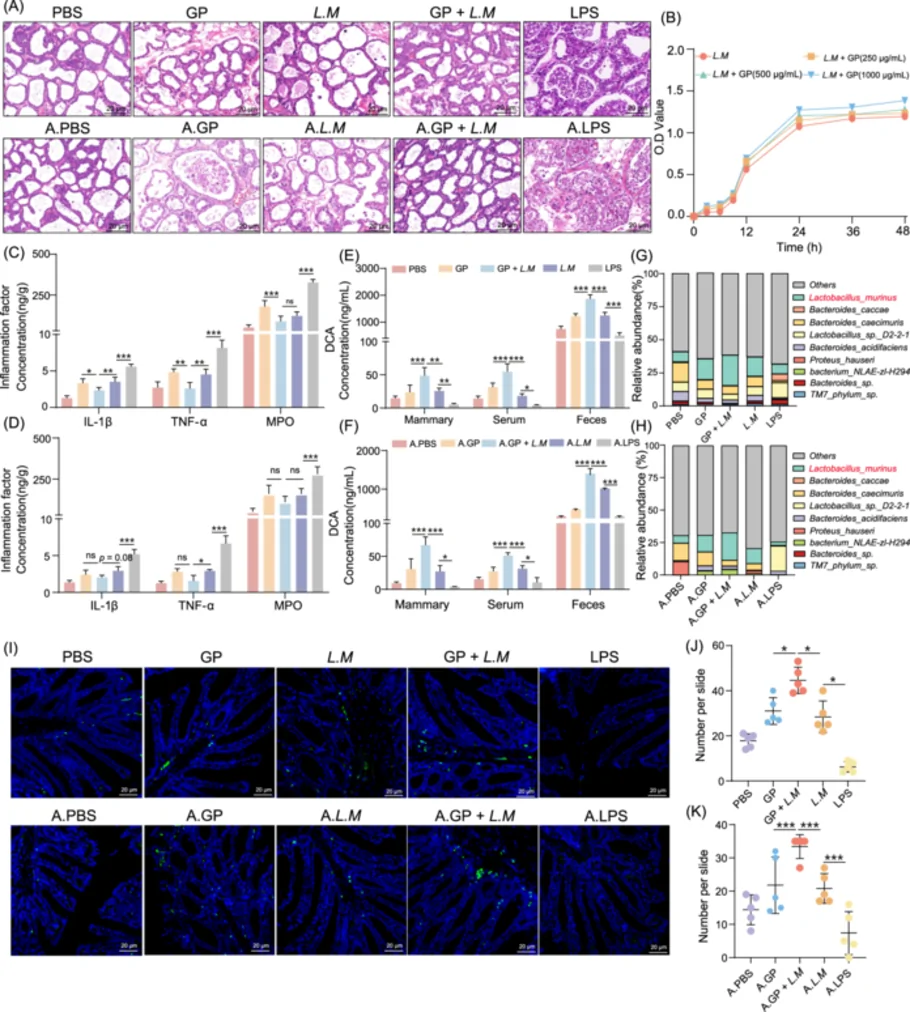
GP‐mediated enrichment of *L. murinus* alleviated LPS‐induced mastitis. (A) Representative H&E staining images of mammary gland. Scale bars: 20 μm. (B) Growth curves of *L. murinus* treated with different GP concentrations. (C, D) MPO, IL‐1β, and TNF‐α in the mammary gland were measured by ELISA kit (*n* = 5). (E, F) DCA in the mammary gland, feces, and serum was measured by ELISA kit. (G, H) Bacterial composition at the species level for the indicated groups was examined (*n* = 5). (I) Fluorescence in situ hybridization (FISH) images of *L. murinus* in colon, blue represents DAPI, green represents *L. murinus*. Scale bar: 20 μm. (J, K) Positive fluorescence count in each group (*n* = 5). All data are expressed as mean ± standard error of the mean (SEM). For the positive fluorescence count of *L. murinu*s detected by FISH, Poisson analysis was used for statistical assessment. A two‐factor model was applied to test the interaction term of GP and *L. murinus* (GP × *L. murinus*). For comparisons among three or more independent groups in a single‐factor design, one‐way ANOVA was conducted, followed by Tukey's post hoc test. For non‐parametric comparison of histological scores among multiple groups, the Kruskal–Wallis test was applied. Statistical significance levels are indicated as follows: *p < 0.05, **p < 0.01, ***p < 0.001. ANOVA, one‐way analysis of variance.

The enhanced therapeutic outcome was mechanistically linked to a marked suppression of local inflammation. Notably, the combination of GP and *L. murinus* exhibited a significantly superior suppressive effect on LPS‐induced pro‐inflammatory cytokines (TNF‐α, IL‐1β, IL‐6) in mammary tissue compared to their individual use, which was further validated by the extremely significant interaction effect between GP and *L. murinus* (*p* < 0.001) (Figure 6C,D). This statistically significant interaction effect confirms that the synergistic suppression of pro‐inflammatory cytokines by GP and *L. M* is not a simple superposition of their individual effects but a result of their positive cooperative interaction, thereby verifying the better therapeutic effect of their combined application. Correspondingly, ELISA analysis revealed that this synergistic effect was associated with a substantial increase in DCA levels in intestinal contents, serum, and mammary glands (Figure 6E,F), reaffirming DCA's central role.

Finally, we investigated the underlying microbial mechanism. Using FISH (Fluorescence in situ hybridization) and 16S rRNA sequencing, we found that co‐administration of GP and *L. murinus* significantly enhanced the colonization of *L. murinus* in the colonic tissues and feces of both mouse models (Figure 6G–K; Figure S5C). The combined treatment also improved overall gut microbiota composition, even in the PGF model (Figure S5D,K).

Collectively, these results demonstrate that GP and *L. murinus* function as an effective synbiotic: GP promotes the proliferation and colonization of the probiotic *L. murinus*, which in turn enhances the production of the therapeutic metabolite DCA, leading to superior protection against LPS‐induced mastitis.

## 3 DISCUSSION

This study provides transformative evidence establishing a causal gut‐mammary axis in mastitis pathogenesis, defines a novel therapeutic pathway mediated by GP, and demonstrates the superior efficacy of a targeted synbiotic as a non‐antibiotic strategy for prevention and treatment of mastitis. While the bile acid–TGR5–cAMP–PKA axis is a recognized anti‑inflammatory signaling framework, our work defines its disease‑specific upstream components within the context of mastitis and maps their functional integration along a newly established gut–mammary circuit.

While associations between gut dysbiosis and mastitis are recognized, establishing definitive causality has been challenging [38]. Utilizing FMT, we demonstrated that microbiota from mastitis mice directly induced mammary inflammation in recipients, accompanied by a dysbiotic gut profile, impaired intestinal barrier, and systemic inflammation. This establishes gut dysbiosis as a driver, not merely a consequence, of mastitis. GP, a major bioactive component categorized as neutral or acidic polysaccharides, demonstrates diverse biological activities such as immune modulation, anti‐inflammatory effects, and gut microbiota regulation [39, 40, 41]. Its protective effect was abolished in pseudo‐germ‐free mice, confirming that GP acts primarily through the gut microbial community rather than via direct systemic anti‐inflammation. Central to this mechanism was the selective enrichment of *L. murinus*, which led to elevated levels of deoxycholic acid (DCA). DCA bound to the TGR5 receptor on mammary epithelial cells, activating the cAMP‐PKA pathway and subsequently suppressing NF‐κB/NLRP3‐driven inflammation—thereby establishing a functional gut–mammary communication axis. *L. murinus* produces various metabolites, including short‐chain fatty acids like butyrate, which were elevated by GP and may support gut health (Table S3). However, among these, the secondary bile acid DCA was the most strikingly elevated and was identified as the central mediator of protection, acting specifically via the mammary TGR5/cAMP‐PKA pathway to alleviate mastitis.

Although our data and published studies support the role of *L. murinus* in DCA production, direct in vitro evidence—such as culturing *L. murinus* with primary bile acid substrates (e.g., cholic acid) and quantifying DCA output by LC‐MS—would further substantiate its bile acid‐transforming capacity in a defined system. Future work should also directly quantify the expression and activity of bile acid‐transforming enzymes (e.g., bile salt hydrolase) in *L. murinus* isolates from mastitis models to fully establish the enzymatic link. Synbiotics often outperform individual probiotics by enhancing probiotic survival, providing rapid activation fuel, and nourishing beneficial bacteria [42, 43]. As expected, the combination of GP and *L. murinus* acted synergistically as a synbiotic, offering superior protection against mastitis compared to either component alone. This synergy likely stems from GP promoting *L. murinus* growth and enhancing its DCA‐producing capacity, while also fostering a more balanced gut microbial environment. As a non‐antibiotic strategy, this synbiotic approach holds promise for mastitis management, particularly during lactation when conventional antibiotics may disrupt maternal and offspring microbiomes [44, 45].

Several limitations of this study merit careful consideration. While our in vitro siRNA knockdown data support the necessity of TGR5 in mammary epithelial cells, the use of *Tgr5*‐knockout mice would provide more definitive in vivo evidence for the receptor's role in mastitis protection. In addition, in conjunction with pharmacological inhibition evidence reported in literature [32, 46], support a causal role for *L. murinus*‐derived DCA in the observed effects, future research—such as culturing *L. murinus* with primary bile acid substrates and quantifying DCA output in a defined system, or leveraging isogenic mutant strains deficient in bile acid‐transforming enzymes (e.g., *bsh*‐knockout *L. murinus*)—would provide more direct biochemical and genetic evidence to validate the bacterial metabolic contribution to mastitis protection. The mechanisms underlying GP's selective enrichment of *L. murinus* remain to be fully elucidated, and potential contributions from other GP‑modulated bacteria or metabolites cannot be excluded. Finally, before clinical translation, the efficacy and safety of GP, *L. murinus*, or their synbiotic formulation require rigorous evaluation in well‑designed trials involving human lactational mastitis and bovine mastitis.

## 4 CONCLUSION

In conclusion, this study causally links gut dysbiosis to mastitis development via the gut‐mammary axis, validated through FMT. We delineate a novel mechanism where the prebiotic GP alleviates mastitis by enriching gut *L. murinus*, thereby increasing its anti‐inflammatory metabolite DCA. DCA signals via mammary epithelial TGR5 receptors, activating the cAMP‐PKA pathway to suppress NF‐κB/NLRP3‐driven inflammation. The synergistic effect of GP and *L. murinus* underscores the significant promise of this synbiotic strategy. Targeting the gut‐mammary axis, particularly the identified microbial (*L. murinus*) and metabolic (DCA‐TGR5‐cAMP‐PKA) pathways, holds significant potential for innovative non‐antibiotic mastitis prevention and treatment.

## 5 METHODS

### Materials

The following reagents were obtained from commercial suppliers: Vancomycin, Penicillin, Metronidazole, and Neomycin (Sigma Aldrich); MDL12330A and H89 (MedChemExpress); and Deoxycholic acid (DCA, Shanghai Yuanye Bio‐Technology Co., Ltd.). Primary antibodies against Claudin‐3 (1:1000; #AF0129; RRID: AB_2833313) were from Affinity Biosciences, while p‐65 (1:1000; #8242), p‐IκBα (1:1000; #2859), PKA (1:1000; #5842), and β‐actin (1:1000; #4970) were sourced from Cell Signaling Technology (CST). Additional antibodies, including Phosphorylation‐p65 (1:1000; GB113882), IκBα (1:1000; GB151509), NLRP3 (1:1000; GB114320), Occludin (1:1000; GB111401), and ZO‐1 (1:1000; GB111402), were purchased from Wuhan Servicebio Technology Co., Ltd. Phosphorylation‐PKA (1:1000; AP0557) was acquired from ABclonal Technology Co., Ltd. Anti‐mouse IgG and anti‐rabbit IgG secondary antibodies were provided by CST. For cytokine quantification, ELISA kits for mouse TNF‐α (EK0527), IL‐1β (EK0394), and MPO (EK0943) were obtained from BOSTER Biological Technology Co., Ltd. LPS and Lipocalin‐2 detection kits were supplied by Shanghai Lanpai Biotechnology Co., Ltd., whereas the DCA ELISA kit was from CLOUD‐CLONE Corp. Wuhan Co., Ltd. siRNA‐TGR5 was custom‐synthesized by JTSBIO Co., Ltd. *Lactobacillus murinus* (American Type Culture Collection, ATCC 35020).

### Preparation and characterization of GP

Dehydrate ginseng roots (1.0 kg) in 50% ethanol for 48 h, and then dehydrate them in 95% ethanol for another 48 h. Subsequently, these slices were soaked twice in deionized water at a weight ratio of 1:10, each time at 100°C for 3 h. Then, all extracts were combined, concentrated with four times the volume of anhydrous ethanol, and allowed to precipitate overnight. After centrifugation at 4000 rpm for 10 min, crude polysaccharides were obtained and deproteinized using Sevag reagent. The resulting polysaccharide solution was further purified by gel‑filtration chromatography on a Sephadex G‑100 column (2.6 × 100 cm) equilibrated with 0.1 M NaCl. The major polysaccharide fraction was collected, dialyzed (molecular weight cut‑off 8000 Da) against distilled water, and lyophilized to obtain the purified GP (>90%).

### Analysis of monosaccharide composition

Mixed standard solutions of different concentrations and standards based on the concentration gradient were prepared previously. For polysaccharide analysis, 5 mg of the sample was accurately weighed and hydrolyzed in 1 mL of 2 M trifluoroacetic acid (TFA) at 121°C for 2 h. Excess acid was removed via nitrogen evaporation and methanol washes, followed by dissolution in sterile water and transfer to a chromatography vial. For chromatographic analysis, an aliquot of the monosaccharide standard solution or polysaccharide hydrolysate was derivatized with 1‐phenyl‐3‐methyl‐5‐pyrazolone (PMP) to enhance separation on the chromatographic column. Chromatographic analysis was then performed using the ThermoU3000 liquid chromatography system, equipped with a ZORBAX Eclipse XDB‐C18 column, and utilizing acetonitrile and phosphate buffer (12 g/L potassium dihydrogen phosphate, adjusted to pH 6.8 with 2 M NaOH) as the mobile phases. The analysis was conducted at specific flow rates, column temperatures, and detection wavelengths.

### Fourier transform infrared spectroscopy analysis

Weigh 1–2 mg of each dried polysaccharide sample in a mortar, add 200 mg of potassium bromide (KBr) powder, and grind evenly. Press the sample into pieces, and use a Fourier transform microscopy infrared spectrometer to scan the sample in infrared with a wavelength range of 4000 to 400 cm^−1^. Record the infrared spectrum.

### Scanning electron microscopy (SEM)

To evaluate the molecular morphology of GP, SEM was employed. The freeze‐dried GP sample was securely positioned on an aluminum plate using double‐sided tape, and a vacuum sputtering coating machine was utilized to deposit a thin layer of gold powder onto the aluminum plate. Subsequently, under high vacuum conditions, the GP samples were analyzed via SEM at an acceleration voltage of 3.0 kV.

### Methylation analysis of GP

One milligram sample was dissolved in DMSO and treated with NaOH. 30 mg of NaOH powder was added and stirred at room temperature for 30 min. Then, 250 μL of CH_3_I was added in five batches and stirred at room temperature for 2 h. Finally, the reaction was terminated by adding 1 mL of water. 2 mL of dichloromethane was added for extraction, and the dichloromethane layer was washed three times with water. Then suck the lower dichloromethane phase and blow dry with nitrogen, adding 1 ml of 2 M TFA and react at 121°C for 2 h. Add 1 M NaBD4 and stir for 2.5 h, then terminate with 300 μL of acetic acid and dry with nitrogen. Add 1.5 mL of acetic anhydride, vortex and mix well, and react at 100°C for 2.5 h. Add 2 mL of water and let it stand for 10 min, then add 1 mL of dichloromethane, vortex and mix well, centrifuge, and discard the aqueous phase. Repeat washing with water three times, take the lower layer of dichloromethane and analyze using a GC‐MS system (Agilent 7890B–5977B, Agilent Technologies).

### Nuclear magnetic resonance (NMR) spectroscopy

Freeze‐dried samples of GP (20.0 mg) were dissolved in 0.5 mL of deuterium oxide (D_2_O; 99.8%; Sigma). The solution was then analyzed using a Bruker Avance 500 MHz nuclear magnetic resonance spectrometer at 60°C with one‐dimensional NMR (1D‐NMR: 1H,13C), and two‐dimensional NMR (2D‐NMR: 1H–1H COSY, HSQC) spectra.

### Untargeted metabolomics

Sixty micrograms of intestinal content was homogenized with 200 μL of extraction solution (methanol:acetonitrile = 1:1, v/v, containing an isotope‐labeled internal standard mixture) by vortexing for 30 s. The mixture was then subjected to ultrasound‐assisted extraction in an ice‐water bath for 10 min. Following extraction, the sample was incubated at −40°C for 1 h to facilitate protein precipitation and then centrifuged at 12,000 rpm (4°C) for 15 min. The resulting supernatant was carefully collected and transferred to a sample vial for subsequent LC‐MS/MS analysis.

Liquid chromatography separation was conducted using a Waters ACQUITY UPLC BEH Amide column. The mobile phase was composed of two buffers: (A) water containing 25 mM ammonium acetate and 25 mM ammonium hydroxide, and (B) acetonitrile. Sample detection was performed using an Orbitrap Exploris 120 mass spectrometer under electrospray ionization conditions set at 3.8 kV for positive mode and −3.4 kV for negative mode. Following the acquisition of raw data, outliers were filtered using the coefficient of variation (CV), and data normalization was applied. Quality control was assessed based on the PCA results derived from quality control (QC) samples. Subsequent data analysis was carried out after these preprocessing steps. Metabolite identification was achieved by referencing the Human Metabolome Database (HMDB) and the Kyoto Encyclopedia of Genes and Genomes (KEGG). Microbial metabolites were further screened using the gutMGene database [47].

### Animals

Male and female BALB/C mice (22–24 g, specific pathogen‐free [SPF] grade) were obtained from Dr. Can Biotechnology (Zhejiang) Co., Ltd. The animals were housed under SPF conditions with ad libitum access to standard diet and water, maintained on a 12‐h light/dark cycle. Following a 1‐week acclimatization period, mice were randomly assigned to cages and mated at a 3:1 male‐to‐female ratio. Because GP treatment was initiated after parturition and the three dams co‑housed in the same cage often gave birth at different times, their respective gavage schedules—and therefore final treatment assignments—were not synchronized. Consequently, in most cages, the co‑housed mice received different experimental treatments (e.g., one might receive GP while another served as a control). This unintended housing pattern meant that mice from different experimental groups shared the same cage‑specific microbial environment, thereby reducing the potential influence of cage‑to‑cage variation on the observed microbiome outcomes.

### Establishment of mouse mastitis model

For seven consecutive days, the NC (normal control) and LPS groups received equal volumes of normal saline via oral gavage, while the GP (treatment) group was administered test samples orally. On Day 8, NC group mice underwent ether inhalation anesthesia. Two hours prior to mastitis induction, mice received intraperitoneal injections of either DCA (10, 20, or 30 mg/kg) or DEX (5 mg/kg). For model establishment, anesthetized female mice were positioned supinely with their fourth mammary glands exposed and sterilized. Using a micro‐syringe, 50 μL LPS (0.2 mg/mL) was bilaterally injected approximately 2 mm along the mammary ducts to induce mastitis [48]. The NC group received the same volume of PBS. After 24 h of LPS infection, the mice were anesthetized and euthanized.

### Establishment of pseudo‐germ‐free mouse model

After continuous gavage with a combination of antibiotics (ABX) (Ampicillin 100 mg/kg, Vancomycin 50 mg/kg, Metronidazole 100 mg/kg, and Neomycin 100 mg/kg) for 5 days [49]. Mice feces were collected after removing the antibiotics for 1 day to verify whether the model was successfully validated and to proceed with the next experiment was performed.

### Oral *L. murinus* treatment

To assess the therapeutic potential of *L. murinus* in LPS‐induced mastitis, mice were divided into five experimental groups: (1) PBS control (PBS); (2) LPS challenge (LPS); (3) *L. murinus* pretreatment (10⁹ CFU/animal/day for 7 days) before LPS challenge (L.M); (4) Combined GP (100 mg/kg) and *L. murinus* (10⁹ CFU/animal/day) pretreatment for 7 days (GP + L.M); and (5) GP alone (100 mg/kg/animal/day) pretreatment for 7 days (GP). Following the treatment period, all animals were humanely euthanized under anesthesia. Blood, serum, mammary tissue, and colon contents were aseptically collected and immediately stored at −80°C for subsequent analyses.

### FMT treatment

The FMT model was established based on the previous study [10]. Fecal suspensions were prepared by homogenizing lyophilized samples (from all experimental groups, including GP.100 and LPS) in sterile PBS (50 mg/mL final concentration). Following centrifugation (100 × *g*, 4°C, 2 min), supernatants were aliquoted for subsequent use. For pseudo‐germ‐mouse colonization, recipient animals received 300 μL of processed microbiota suspension daily for three consecutive days after antibiotic withdrawal (initiated 1‐week post‐pregnancy). This was followed by biweekly administrations for 3 weeks to maintain stable colonization.

### Cell culture and treatment

HC11(STCC20048P, Wuhan Servicebio Technology Co., Ltd.) mouse mammary epithelial cells were cultured in DMEM supplemented with 10% fetal bovine serum and 1% penicillin/streptomycin at 37°C with 5% CO₂. For DCA treatment, cells were seeded in six‐well plates at 10⁶ cells/mL for 24 h, then pretreated with MDL12330A (10 μM) or H89 (30 μM) for 2 h before adding DCA (10–30 μM) 2 h prior to LPS stimulation. For siRNA experiments, cells were transfected with 50 nM TGR5 or control siRNA using Lipofectamine 3000 in Opti‐MEM, incubated in serum/antibiotic‐free medium for 6 h, then returned to complete medium. Forty‐eight hours post‐transfection, cells were treated with DCA for 2 h followed by LPS stimulation (1 μg/mL) for 6 h before harvest.

### Gut microbiota analysis

Fecal samples were collected from each mouse, and genomic DNA was extracted using the ALFA‐SEQ Advanced Soil DNA Kit (MP Biomedicals). The V3‐V4 hypervariable region of bacterial 16S rRNA was amplified by PCR with universal primers 338F (ACTCCTACGGGAGGCAGCA) and 806R (GGACTACHVGGGTWTCTAAT). Each 50 µL reaction contained 1 µL of template DNA (approximately 50 ng), 1 µM of each primer, and 25 units of TAKKARA Taq DNA polymerase. The thermal cycling conditions were initially denaturation at 94°C for 5 min; 32 cycles of 94°C for 30 s, 52°C for 30 s, and 72°C for 30 s; and a final extension at 72°C for 10 min. The fragment length and concentration of PCR products were determined by 1% agarose gel electrophoresis and set a negative control to ensure the quality of DNA. The Sequencing libraries were constructed according to the standard process of the ALFA‐SEO DNA Library Prep Kit, and the size of the library fragments was evaluated using the Qsep400 high‐throughput nucleic acid protein analysis system (Hangzhou Houze Biotechnology Co., Ltd.). The concentration of the library was measured using Qubit 4.0 (Thermo Fisher Scientific). The double‐ended sequencing was performed on the Illumina or MGI PE250 platform (Illumina).

To integrate the raw double‐end sequencing data, paired‐end raw reads were filtered using fastp (version 0.14.1, https://github.com/OpenGene/fastp) with a sliding window quality trimming setting (‐W 4 ‐M 20). Primers were subsequently removed with cutadapt (https://github.com/marcelm/cutadapt/), yielding high‐quality paired‐end clean reads. These clean reads were then merged using usearch ‐fastq_mergepairs (V10, http://www.drive5.com/usearch/), with parameters set to a minimum overlap length of 16 bp and a maximum of five mismatches in the overlap region. The merged sequences, termed raw tags, were further quality filtered using fastp (‐W 4 ‐M 20) to produce clean tags. Clean tags that met the quality control criteria were then clustered into amplicon sequence variants (ASVs) using the QIIME2 bioinformatics pipeline [50]. All clean tags obtained after merging and filtering were clustered and assigned using SILVA reference (version 138) database, while simultaneously removing chimeras by USEARCH (V10.0.240) [51]. In analyses focused on classified taxa, unclassified bacteria were excluded to improve the interpretability of taxonomic shifts. Alpha diversity was calculated based on ASVs abundance (using Chao, Simpson, and Shannon indices) using USEARCH (‐alpha_div, V10, http://www.drive5.com/usearch/) and the vegan package (version 2.5‐3) in R software. PCoA based on Bray–Curtis distance was used to determine beta diversity, which was performed to assess the relationships between different groups, using vegan package (version 2.5‐3) in R software. Finally, LEfSe analysis (LDA > 3, with FDR‐corrected *p*‐values) was conducted to identify bacterial taxa that causes differences between groups, using the Galaxy platform for analysis.

### Histological analysis and immunohistochemical staining

Tissue samples were fixed in 4% paraformaldehyde (≥48 h), paraffin‐embedded, and sectioned at 5 μm thickness. Following standard dewaxing and hydration procedures, sections were stained with H&E for microscopic evaluation. Histopathological alterations were assessed by light microscopy and quantified using standardized scoring criteria [52]. MUC2 expression was evaluated by immunohistochemistry (IHC) using optical microscopy (Olympus).

### Fluorescence in situ hybridization (FISH)

The probe sequence (5'‐TTACTCTCACGCACTTTCTTCTCTAACAACAG‐3') was derived from the 16S rRNA of *L. murinus*, and its specificity was verified in silico against the SILVA database. In analyses focused on classified taxa, unclassified bacteria were excluded to improve the interpretability of taxonomic shifts. Fixed colon samples were pre‐hybridized in humid chambers at 38–42°C for 2–4 h, followed by overnight hybridization with the probe solution under the same temperature conditions. Post‐hybridization washes were performed sequentially with 2×, 0.5×, and 0.2× SSC buffers at 37°C. After blocking, samples were incubated with HRP‐conjugated secondary antibodies and visualized by fluorescence microscopy.

### TNF‐α, IL‐1β, MPO, Lipocalin‐2 and DCA detections

Serum and tissue levels of TNF‐α, IL‐1β, MPO, and DCA were quantified using ELISA. Mammary gland and colon tissues were processed by homogenization in 10% PBS followed by centrifugation (12,000 × *g*, 10 min) to obtain supernatants. Similarly, fecal samples were weighed, homogenized in 10% PBS, and centrifuged (3000 × *g*, 4°C, 10 min) for supernatant collection.

### Quantitative real‐time PCR (RT‐qPCR)

Total RNA was isolated from cultured cells (six‐well plates) using 1 mL Trizol reagent, with concentration quantified by Nanodrop One (Thermo Fisher Scientific). Reverse transcription was performed using a commercial cDNA synthesis kit (Yeasen Biotechnology), followed by qPCR with SYBR Green Master Mix. Gene expression levels were normalized to β‑actin and calculated using the 2^−ΔΔCt^ method. Primer sequences are listed in Table S1.

### Alcian blue (AB) staining

Colon tissue sections were deparaffinized through xylene and graded alcohol series, followed by mucin‐specific staining using an Alcian Blue Stain Kit (Solarbio) according to the manufacturer's protocol.

### Serum biochemical examination

Blood samples from FMT‐treated mice were collected in thrombin‐activated vacuum tubes and analyzed using an automated biochemical analyzer (BS240).

### Western blot assay

Total protein was extracted from precisely weighed mammary gland and colon tissue samples (approximately 50–100 mg per sample) using ice‐cold RIPA lysis buffer supplemented with 1% protease inhibitor cocktail (PMSF). The homogenates were then centrifuged at 12,000 × *g* for 10 min at 4°C to obtain clear protein supernatants. Protein quantification was performed using a BCA protein assay kit according to the manufacturer's protocol, with bovine serum albumin (BSA) as the standard. Protein samples (20–40 μg per lane) were mixed with 5× SDS loading buffer, denatured at 95°C for 5 min, and separated by 10–12% SDS‐PAGE at 80–120 V for 90–120 min. The separated proteins were electrophoretically transferred to PVDF membranes (0.45 μm pore size). Membranes were then blocked with 5% (w/v) non‐fat dry milk in TBST (Tris‐buffered saline with 0.1% Tween‐20) for 2 h at room temperature with gentle agitation. Primary antibody incubation was performed overnight at 4°C in blocking buffer at optimal dilutions (1:1000). After extensive washing (3 × 5 min with TBST), membranes were incubated with HRP‐conjugated secondary antibodies (1:5000 dilution) for 2 h at room temperature. Following additional TBST washes, protein bands were visualized using enhanced chemiluminescencereagent and imaged plus Western Blotting Detection system.

### Immunofluorescence assay

Tissue sections were processed through xylene dewaxing and graded ethanol dehydration, followed by permeabilization with 0.3% Triton X‐100 (Sigma) in PBS containing 10% BSA. Primary antibodies were applied overnight at 4°C, with species‐matched secondary antibodies subsequently incubated for 1 h at room temperature. Nuclei were counterstained with DAPI (4',6‐Diamidino‐2‐Phenylindole) before fluorescence microscopic analysis.

### Statistics

Statistical analyses were conducted using GraphPad Prism 8.0.2 (GraphPad Software). Experimental data were presented as the mean ± standard error of the mean (SEM) or by boxplots. For comparisons between two independent groups, statistical significance was determined using an unpaired two‐tailed Student's *t*‐test. One‐way analysis of variance (ANOVA) followed by Tukey's post hoc test was applied for multiple comparisons among three or more independent groups. The Kruskal–Wallis test was used for non‐parametric statistical comparisons of semi‐quantitative scores across multiple groups. Poisson regression analysis was performed for the statistical evaluation of count data, and a two‐factor ANOVA model was adopted to examine the interaction effect between two experimental factors. Statistical significance was defined as follows: *p < 0.05, **p < 0.01, ***p < 0.001.

## AUTHOR CONTRIBUTIONS

**Qi Su** and **Haichong Wu**: Conceptualization; supervision. **Zhijie Zheng, Sizhe Chen, Huiying Liu, Jianhui Gu, Sisi Cai**, and **Xiang Liu**: Investigation. **Zhijie Zheng, Sisi Cai, Raphaela I Lau, Yao Zhang**, and **Xiaoyan Wang**: Formal analysis. **Zhijie Zheng, Sisi Cai, Renyou Gan, Haichong Wu, Kewei Fan, Hein Min Tun, Sunjae Lee**, and **Xiaole Yin**: Writing—original draft; writing—review and editing. All authors have read the final manuscript and approved it for publication.

## CONFLICT OF INTEREST STATEMENT

The authors declare no conflicts of interest.

## ETHICS STATEMENT

All mouse maintenance and experimental procedures were performed in strict accordance with the guidelines approved by the Lab of Animal Experimental Ethical Inspection of Dr. Can Biotechnology (Zhejiang) Co., Ltd. (Approval No.: DRK‐2023072598541, DRK‐202311099245, DRK‐2023120185469, and DRK‐2025010382541r).

## Supporting information


**Figure S1.** The gut microbiota is closely linked to mastitis and induce intestinal barrier injury and mammary gland inflammation.
**Figure S2.** Structural analysis of ginseng polysaccharides.
**Figure S3.** Ginseng polysaccharides improve mastitis through gut microbiota mediated NF‐κb/NLRP3 signaling pathway.
**Figure S4.** DCA reduced the inflammatory response of MMECs to LPS *via* cAMP‐PKA‐NF‐κB/NLRP3 pathway mediated by TGR5.
**Figure S5.** Changes in gut microbiota structure of mice after oral administration of *Lactobacillus murinus* and GP in SPF and PGF mice.

**Table S1.** The oligonucleotides used in this study.
**Table S2.** Analysis results of sugar residues in GP.
**Table S3.** SCFAs concentrations in GP.100 and LPS groups.

## Data Availability

Sequencing data that support the findings of this study were publicly available in NCBI under the BioProject ID of PRJNA1110719 (https://www.ncbi.nlm.nih.gov/bioproject/?term=PRJNA1110719) and PRJNA1251275 (https://www.ncbi.nlm.nih.gov/bioproject/?term=PRJNA1251275). The data used are saved in GitHub https://github.com/ZhijieZheng-magic/iMetaOmics. Supplementary materials (figures, tables, graphical abstract, slides, videos, Chinese translated version, and update materials) may be found in the online DOI or iMeta Science http://www.imeta.science/imetaomics/.
