# Supplementary material for: Ginseng polysaccharides prevent mastitis through Lactobacillus murinus‐derived deoxycholic acid and TGR5 signaling
Source: IMetaOmics. 2026 Mar 3;3(2):e70090. doi: 10.1002/imo2.70090 (PMC13674426; doi:10.1002/imo2.70090)
Supplement: Supplementary file 1 [file IMO2-3-e70090-s001.docx]

Supporting information to

**Ginseng Polysaccharides Prevent Mastitis Through *Lactobacillus murinus*-Derived Deoxycholic Acid and TGR5 Signaling**

Zhijie Zheng^1,2,3#^, Huiying Liu^4#^, Sizhe Chen^2,^^3#^, Jianhui Gu^5^, Sisi Cai^1^, Xiang Liu^1^, Raphaela I Lau^2,3^, Yao Zhang^6^, Sunjae Lee^7^, Xiaoyan Wang^1,8^, Kewei Fan^9^, Xiaole Yin^10,11^, Hein Min Tun^2,12,13^, Renyou Gan^13,14,15^, Haichong Wu^1,10*^, Qi Su^2,3*^

^1^MOA Key Laboratory of Animal Virology, Zhejiang University Center for Veterinary Sciences, Hangzhou 310058, China.

^2^Microbiota I-Center (MagIC), Hong Kong, China.

^3^Department of Medicine and Therapeutics, State Key Laboratory of Digestive Diseases, Li Ka Shing Institute of Health Sciences, The Chinese University of Hong Kong, Hong Kong, China.

^4^Key Laboratory of Tumor Microenvironment and Immune Therapy of Zhejiang Province, The Second Affiliated Hospital, Zhejiang University School of Medicine, Hangzhou 310009, China.

^5^State Key Laboratory of Plant Genomics, Institute of Genetics and Developmental Biology (IGDB), Chinese Academy of Sciences, Beijing 100101, China.

^6^Department of Gastroenterology, Ruijin Hospital Affiliated to Shanghai Jiao Tong University School of Medicine, Shanghai 200025, China.

^7^ School of Life Sciences, Gwangju Institute of Science and Technology, Gwangju 61005, Republic of Korea.

^8^College of Animal Science and Technology, Chongqing Three Gorges Vocational College, Chongqing 404155, China.

^9^Fujian Provincial Key Laboratory for the Prevention and Control of Animal Infectious Diseases and Biotechnology, College of Life Sciences, Longyan University, Longyan 364012, China.

^10^School of Civil and Environmental Engineering, Nanyang Technological University, Singapore 639798, Singapore.

^11^Channing Division of Network Medicine, Department of Medicine, Brigham and Women's Hospital, Harvard Medical School, Boston, Massachusetts 02115, United States.

^12^The Jockey Club School of Public Health and Primary Care, Faculty of Medicine, The Chinese University of Hong Kong, Hong Kong, China.

^13^Li Ka Shing Institute of Health Sciences, Faculty of Medicine, The Chinese University of Hong Kong, Hong Kong, China.

^14^Department of Food Science and Nutrition, The Hong Kong Polytechnic University, Hong Kong, China.

^15^Research Institute for Future Food, The Hong Kong Polytechnic University, Hong Kong SAR, China.

^#^These authors contributed equally: Zhijie Zheng, Huiying Liu and Sizhe Chen.

^*^Correspondence: [haichongwu@zju.edu.cn](mailto:haichongwu@zju.edu.cn) (Haichong Wu), [qisu@cuhk.edu.hk](mailto:qisu@cuhk.edu.hk) (Qi Su)

**Figure S1 The gut microbiota is closely linked to mastitis and induce intestinal barrier injury and mammary gland inflammation.** (A) Representative H&E staining images of mammary gland. Scale bars: 20 μm. The arrow points to the infiltration of inflammatory cells and the disruption of the mammary gland acinar structure. (B) The bacterial composition at the Genus level was examined for the indicated groups. (C) PCoA analysis was conducted to measure the changes in the composition of the gut microbiota in NC and A-NC group mice (*p* = 0.002, *R^2^* = 0.5994, F = 14.965). The results showed significant differences in gut microbiota between ABX treated mice and NC mice. (D-F) The α diversity index includes Richness, Chao1, and Shannon_e were detected in NC and A-NC group mice (*n* = 5). (G) The bacterial composition at the genus level was examined for the indicated groups. (H) Representative western blot images of inflammatory-related proteins and tight junction proteins in mammary gland. (I) Proteins level of Western blot was measured using Image J (*n* = 3). The statistical differences of all groups were compared with the LPS group. (J-K) Protein levels of colonic ZO-1, Occludin, and Claudin-3 were measured using western blot (*n* = 3). All data are presented as the mean ± SEM. For comparisons between two groups, statistical significance was assessed using an unpaired two-tailed Student’s *t*-test. For comparisons among three or more independent groups in a single-factor design, a one-way analysis of ANOVA was performed; For non-parametric comparison of histological scores among multiple groups, the Kruskal–Wallis test was applied. Statistical significance was indicated as follows: **p* < 0.05, ***p* < 0.01, ****p* < 0.001.

**Figure S2 Structural analysis of ginseng polysaccharides.** (A) FTIR spectrum. (B) SEM images of GP. Scale bar, 50 μm (up) and 10 μm (down). (C) Monosaccharide standards. (1: Mannose; 2: Glucosamine hydrochloride; 3: Rhamnose; 4: Glucuronic Acid; 5: Galacturonic Acid; 6: Galactosamine hydrochloride; 7: Glucose; 8: Galactose; 9: Xylose; 10: Arabinose; 11: Fructose. (D) Monosaccharide of GP. (E)^1^H NMR. (F) ^13^C NMR. (G) ^1^H-^1^H COSY. (H) HSCQ. (I) NOESY. (J) HMBC and (K) HSQC-TOCSY. (L) The structure of GP.

**Figure S3 Ginseng polysaccharides improve mastitis through gut microbiota mediated NF-κb/NLRP3 signaling pathway.** (A) MPO, IL-1β, and TNF-α in the mammary gland were measured by ELISA kit (*n* = 5). The statistical differences of all groups were compared with the LPS group. (B-C) Representative western blot images of inflammatory-related proteins and measured using Image J (*n* = 3). The statistical differences of all groups were compared with the LPS group. (D-E) Protein levels of ZO-1, Occludin, and Claudin-3 in mammary gland were measured using western blot (*n* = 3). The statistical differences of all groups were compared with the LPS group. (F) Bacterial composition at the Phylum level for the indicated groups was examined (*n* = 5). (G-H) Shannon_e, and Chao1 index were measured in each group (*n* = 5). (I-J) *Firmicutes* and *Firmicutes*/*Bacteroidota* ratio in each group (*n* = 5). All data are presented as the mean ± SEM. For comparisons between two groups, statistical significance was assessed using an unpaired two-tailed Student’s *t*-test. For comparisons among three or more independent groups in a single-factor design, a one-way analysis of ANOVA was performed. Statistical significance was indicated as follows: **p* < 0.05, ***p* < 0.01, ****p* < 0.001.

**Figure S4 DCA reduced the inflammatory response of MMECs to LPS *via* cAMP-PKA-NF-κB/NLRP3 pathway mediated by TGR5.** (A-D) Cells were pretreated with DCA (10,20,30 μM), and the relative protein levels of cAMP-PKA-NF-κB/NLRP3 pathway were detected by Western blotting. Representative western blot images of proteins in the mammary gland were measured using ImageJ (*n* = 3). (E-H) Cells were pretreated with H89 the relative protein levels of cAMP-PKA-NF-κB/NLRP3 pathway were detected by Western blotting. Representative western blot images of proteins in the mammary gland were measured using ImageJ (*n* = 3). (I-L) Cells were pretreated with siRNA-TGR5, MDL12330A, and H89 and the relative protein levels of cAMP-PKA-NF-κB/NLRP3 pathway were detected by Western blotting. Representative western blot images of proteins in the mammary gland were measured using ImageJ (*n* = 3). All data are presented as the mean ± SEM. For comparisons among three or more independent groups in a single-factor design, a one-way analysis of ANOVA was performed. Statistical significance was indicated as follows: **p* < 0.05, ***p* < 0.01, ****p* < 0.001.

**Figure S5 Changes in gut microbiota structure of mice after oral administration of *Lactobacillus murinus* and GP in SPF and PGF mice.** (A-B) Mammary gland histological scores were determined based on H&E-stained sections (*n* = 5). (C) LEfSe analysis was conducted to identify the different bacterial taxa enriched in different groups (log_10_LDA score > 2). (D) PCoA analysis was conducted to measure the changes in the composition of the gut microbiota in each group of pseudo sterile mice (*p* = 0.089, *R^2^* = 0.1914, F = 1.8215). (E-G) Richness, Shannon_e, and Chao1 index in each group of pseudo sterile mice. (H) PCoA analysis was conducted to measure the changes in the composition of the gut microbiota in each group of SPF mice (*p* = 0.001, *R^2^* = 0.2406, F = 2.2022). (I-K) Richness, Shannon_e, and Chao1 index in each group of SPF mice. All data are presented as the mean ± SEM. For comparisons among three or more independent groups in a single-factor design, a one-way analysis of ANOVA was performed. Statistical significance was indicated as follows: **p* < 0.05, ***p* < 0.01, ****p* < 0.001.
